# Spindle Epithelial Tumor With Thymus-Like Differentiation (SETTLE) Misdiagnosed as Papillary Thyroid Carcinoma: A Case Report

**DOI:** 10.7759/cureus.31574

**Published:** 2022-11-16

**Authors:** Kumaresan Kathamuthu, Chandrashekar Janardan, Subramaniam Ramkumar, Lovy Agarwal, Nabeel Al-Brahim, Manal Eldein Ahmed, Jayagandan Jayamani

**Affiliations:** 1 Department of Laboratory Medicine, New Mowasat Hospital, Al-Salmiya, KWT; 2 Department of Anatomic and Clinical Pathology, Woodland Hospital, Shillong, IND; 3 Department of Pathology, Kuwait Cancer Control Centre, Shuwaikh, KWT; 4 Department of Laboratory Medicine, Farwaniya Hospital, Kuwait City, KWT; 5 Department of Laboratory Medicine, New Mosawat Hospital, Al-Salmiya, KWT

**Keywords:** spindle epithelial tumor with thymus-like differentiation (settle), spindle epithelial tumor characterised with thymus-like differentiation, thymoma, synovial sarcoma, papillary thyroid carcinoma

## Abstract

Spindle epithelial tumor with thymus-like differentiation (SETTLE), a rare tumor of the thyroid gland, is difficult to diagnose irrespective of its unique morphology. It is usually misdiagnosed as synovial sarcoma, thymoma, teratoma, or other thyroid carcinomas. In the current case report, we detail a case of a 36-year-old male patient who presented with thyroid swelling that was initially misdiagnosed as papillary thyroid carcinoma instead of SETTLE. Based on fine needle aspiration, the tumor showed a variable pattern with features suggestive of follicular neoplasm in the right lobe and atypia of undetermined significance in the left lobe. Pathological examination showed multiple nodules on both the right and left lobes, with the largest nodule measuring 4.8 x 4.5 x 3 cm. On microscopic examination, a predominant papillary pattern was observed along with spindle cell areas. Immunohistochemistry revealed positive staining for thyroglobulin, CK, HMWCK, CD99, and BCL-2, which led to the diagnosis of SETTLE. The rare nature of the condition and the reduced awareness about it make this tumor a diagnostic challenge. This case report concludes that in case of any biphasic tumor with epithelial and spindle cells in the thyroid gland, it is important to consider the differential diagnosis of SETTLE. Immunohistochemistry is more useful for diagnosing SETTLE, and thus pathologists are encouraged to judiciously advise the patients for immunohistochemistry to establish accurate and efficient diagnosis.

## Introduction

Spindle epithelial tumor with thymus-like differentiation (SETTLE) is a rare malignant thyroid tumor, with a risk of metastatic potential occurring predominantly among children and adolescents. It is believed to have emerged from the remnants of the fourth and fifth branchial pouches or the thymus and is malignant in nature [1]. It is often referred to as a low-grade malignancy due to its delayed metastasis to the lymph nodes or lungs [2].

SETTLE exhibits a biphasic morphology consisting of a mixture of spindle cells predominating over an epithelial component. Although it has a unique morphology, the diagnosis is challenging, and it is often misdiagnosed as a thyroid carcinoma such as spindle cell medullary carcinoma and papillary thyroid carcinoma (PTC). It has previously been published under the titles of “thyroid thymoma,” “malignant teratoma,” and “thyroid spindle cell tumor” [3]. Although surgery is the primary mode of management, chemotherapy and radiotherapy are also used in advanced cases [4].

As a rare entity, SETTLE is often not considered in the differential diagnosis of thyroid malignancies. There is paucity in the literature and no hallmark finding to differentiate SETTLE from other tumors by noninterventional modes of testing such as ultrasonography (USG). Fine needle aspiration (FNAC) biopsy for SETTLE shows variable cytological features and the lack of specificity for diagnosing a case of SETTLE [4]. Moreover, the lack of specific molecular markers for SETTLE makes this ancillary mode of testing unreliable [5]. It is mainly immunohistochemistry (IHC; positivity for cytokeratin (CK) and vimentin and negativity for thyroid markers such as thyroglobulin and TTF1) that provides sufficient evidence to establish a diagnosis of SETTLE [4].

This necessitates a thorough understanding of this rare entity to aid in the accurate diagnosis of the condition and to prevent the misdiagnosis of thyroid carcinomas. In the present case report, we describe a case of a middle-aged male patient with SETTLE that was initially misdiagnosed as PTC. This case provides a detailed discussion of the diagnostic difficulties. The diagnosis of SETTLE requires detailed histology, and IHC is the mainstay of diagnosis. Although a rarity, this report suggests considering SETTLE as a differential diagnosis of PTC especially in the young population presenting with a thyroid mass.

## Case presentation

A 36-year-old male patient presented with a slowly progressing thyroid swelling for more than four months. On clinical assessment, a firm, nontender, palpable mass was observed on the right and left lobes of the thyroid gland. The patient had no medical or family history. USG of the neck revealed an enlarged thyroid gland with a heterogeneous parenchymal echo pattern and multiple variable-sized hypo-/isoechoic nodules seen on both lobes. The largest nodule identified on the right lobe measured 4 × 3.6 cm, and the largest nodule on the left lobe measured 2.8 × 2.5 cm. No enlarged cervical lymph nodes were identified. The patient was further referred for USG-guided FNAC. The aspirate smears on cytopathologic examination from both lobes showed a variable pattern with features suggestive of follicular neoplasm (Bethesda category IV) on the right lobe and features suggestive of atypia of undetermined significance (AUS) on the left lobe (Bethesda category III). Subsequently, the patient underwent total thyroidectomy with no cervical lymph node dissection. Pathological examination showed multiple nodules on both the right and left lobes. The largest nodule on the right lobe, measuring 4.8 × 4.5 × 3 cm, showed a fleshy appearance with areas of hemorrhage. Grossly, no extra thyroidal extension was identified.

**Figure 1:**
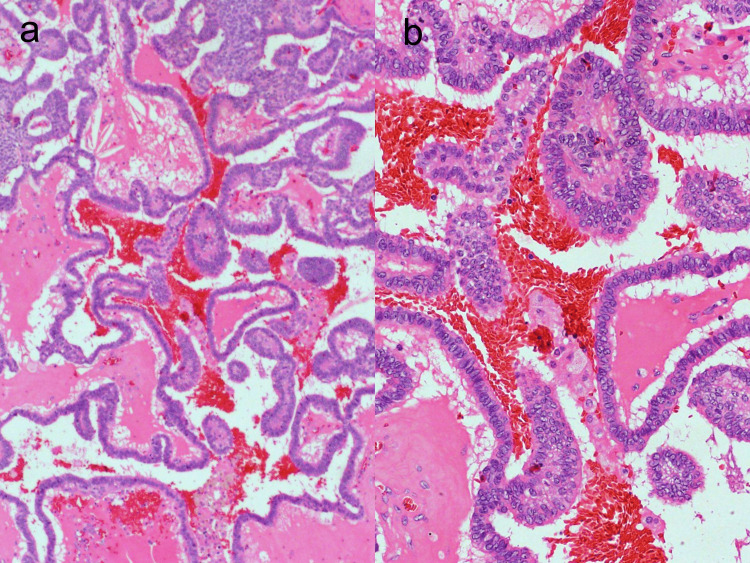
Papillary tumor component seen in a case of SETTLE. (a) H&E x10. (b) H&E x40. SETTLE showing neoplastic epithelial cells arranged in papillary clusters. The cells show increased nuclear cyoplasmic ratio, moderately pleomorphic nuclei, with nuclear crowding, nuclear overlapping, and ground-class chromatin. However, nuclear membrane irregularities, nuclear grooves, and intranuclear cytoplasmic pseudoinclusions were not conspicuous in this case, as seen in the images. SETTLE, spindle epithelial tumor with thymus-like differentiation

Microscopic examination showed an encapsulated biphasic tumor involving the right lobe and consisting of spindle cells and epithelial cells. The epithelial cells were well organized in well-formed papillary structures (Figures 1a, 1b).

**Figure 2:**
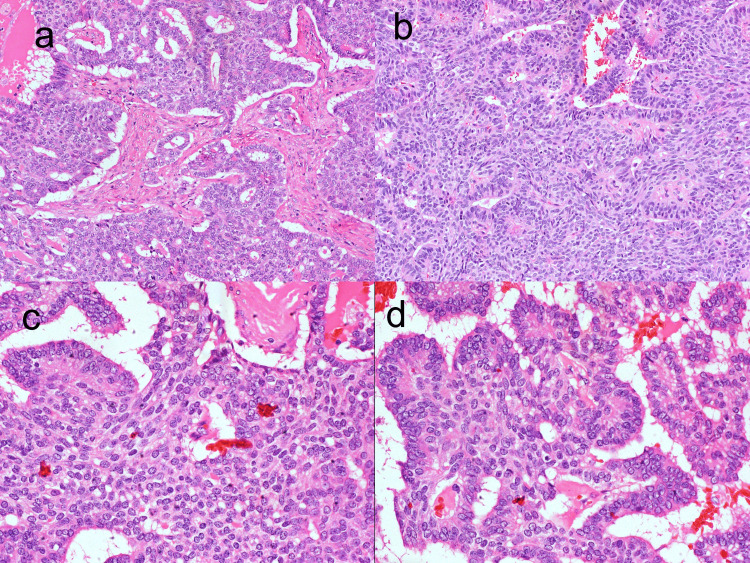
Solid component in a case of SETTLE. (a and b) x10. (c and d) x40. Solid component in a case of SETTLE showing spindle-shaped cells arranged in sheets and nests, resembling a poorly differentiated carcinoma. The cells also show stippled chromatin. SETTLE, spindle epithelial tumor with thymus-like differentiation

Foci of glandular and cribriform pattern were also noted surrounded by solid areas of spindle cells (Figures 2a-2d).

These solid and spindle areas were interpreted as poorly differentiated areas of PTC. Consequently, a diagnosis of PTC was made based on the papillary architecture and the presence of focal nuclear features such as nuclear enlargement, elongation, and chromatin clearing. However, there were no nuclear membrane irregularities such as grooving and nuclear pseudoinclusions. On the basis of the above morphology, a diagnosis of PTC characterized by a focal inadequate differentiated area was made. Subsequently, the patient was directed to visit a regional cancer center for further medical intervention. The slides and blocks were reviewed by an endocrine pathologist, and IHC was conducted.

**Figure 3:**
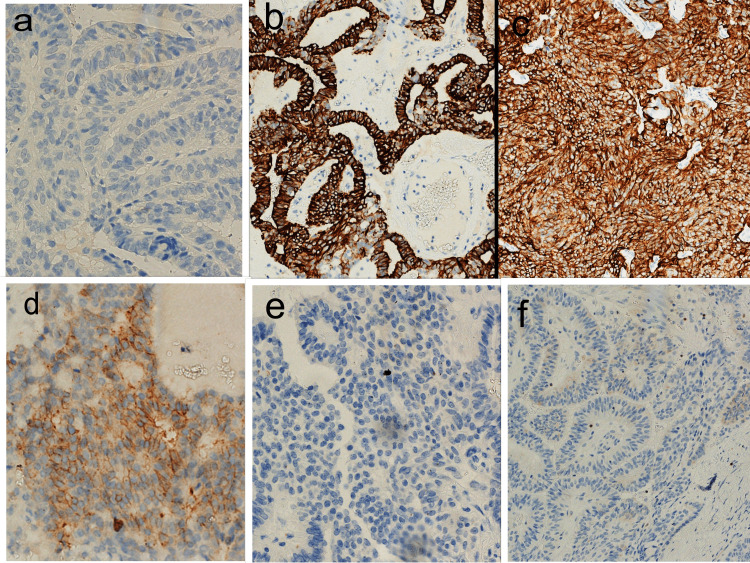
Immunohistochemical panel in a case of SETTLE. (a-c and f) 10x. (d and e) 40x. On immunohistochemistry, the tumor cells showed positive immunostaining for HMWCK (Figures 3b, 3c) and CD99 (Figure 3d), and negative immunostaining for thyroglobulin (Figure 3a), chromogranin (Figure 3e), and Bcl-2 (Figure 3f). SETTLE, spindle epithelial tumor with thymus-like differentiation

On IHC (Table 1), the tumor cells showed positive immunostaining for high molecular weight cytokeratin (HMWCK) (Figures 3b, 3c) and CD99 (Figure 3d), and negative immunostaining for thyroglobulin (Figure 3a), chromogranin (Figure 3e), and Bcl-2 (Figure 3f).

**Table 1:** Details of antibodies and relevant immunohistochemistry protocols used in the study. The sections were deparaffinized and rehydrated. A multi-epitope retrieval system (PathnSitu Biotechnologies, Livermore, CA) was used, and retrieval was done in a citrated buffer (pH 6). The process was performed for five minutes at 120 degree Celsius, followed by cooling for 10 minutes. Immunostaining was done. All tissues were then exposed to 3% H_2_O_2_ for five minutes, a primary antibody for 25 minutes, polyexcel target binder for 10 minutes, polyexcel HRP for 10 minutes, diaminobenzidine as chromogen for five minutes, and hematoxylin as a counterstain for one minute. In between incubations, the sections were sufficiently washed with tris-buffered saline. The incubation procedure was performed at room temperature.

Name of antibody	Source	Clone	Dilution	Name of supplier
Thyroglobulin-2	EP250	Rabbit monoclonal	Ready to use	PathnSitu, Livermore, CA
HMWCK	34BE12	Mouse monoclonal	Ready to use	PathnSitu, Livermore, CA
CD99	EP8	Rabbit monoclonal	Ready to use	PathnSitu, Livermore, CA
Chromogranin	EP38	Rabbit monoclonal	Ready to use	PathnSitu, Livermore, CA
Bcl-2	EP36	Rabbit monoclonal	Ready to use	PathnSitu, Livermore, CA

Final diagnosis of SETTLE was made based on the above combined histomorphological and immunohistochemical findings. The patient has been on regular follow-up and has been asymptomatic with no recurrence to date.

## Discussion

SETTLE was initially reported by Chan and Rosai in 1991 to describe a set of tumors of the thyroid glands that develop as a painless slow-growing mass in the neck region [1]. These tumors have a well-defined capsule and are usually infiltrating in nature. A characteristic feature is their biphasic histomorphology, accompanied by a mixture of ductal, spindle, and epithelial cells (Figures 1, 2). The tumor cells exhibit positivity for CK (Figures 3b, 3c), P40, P63, epithelial membrane antigen (EMA), smooth muscle actin (SMA), and vimentin. These tumor cells are usually negative for thyroid transcription factor-1 (TTF-1), carcinoembryonic antigen (CEA), thyroglobulin (Figure 3a), calcitonin, and chromogranin (Figure 3e) [6-8].

All of these features of SETTLE were consistently observed in our case. Another striking feature observed in our case on IHC was the positivity for HMWCK exhibited by the tumor cells (Figures 3b, 3c) as it may be evidence to suggest an origin from the remnants of the ultimobranchial body. There are a few studies wherein these clinical, histopathological, and immunohistochemical characteristics have been recorded, and the findings from our report are consistent with existing literature [3-7,9].

However, most cases of SETTLE were not easily diagnosed and resulted in misdiagnosis. Differential diagnoses include a variety of biphasic epithelial and spindle head and neck region tumors. Some of them include ectopic spindle cell thymoma, immature teratomas, synovial sarcoma (SS), a spindle cell variant of medullary and papillary carcinoma of the thyroid, solitary fibrous tumor, and sarcomatoid anaplastic thyroid carcinoma. Careful histological and immunohistochemical examinations, as well as the correlation of the clinical and radiological findings, have been helpful in distinguishing and establishing the accurate diagnosis [9].

In our study, the patient was misdiagnosed to have PTC. This was a provisional diagnosis made before confirming the diagnosis with IHC as a case of SETTLE. Studies have shown that PTC is the most prevalent type of tumor of the thyroid glands, constituting approximately 80% of all thyroid malignancies [10]. It predominantly affects the thyroid gland and has approximately 15 variants. PTC is usually diagnosed with reference to the nuclear features. The tumor is characterized by changes in nuclear size and shape and changes in chromatin characteristics. The changes in nuclei include nuclear membrane irregularity, nuclear overlapping, enlargement, and elongation. Conversely, changes in chromatin characteristics include glassy nuclei, chromatin clearing, and margination.

In our case, a diagnosis of PTC was determined based on the papillary architecture and occurrence of focal nuclear features such as nuclear enlargement, elongation, and chromatin clearing (Figure 1). However, no nuclear membrane irregularities such as grooving and nuclear pseudoinclusions were observed. Two populations of tumor cells consisting of spindle and epithelial cells were observed (Figures 1, 2). The epithelial cells were organized in a papillary pattern. Conversely, the spindle cells were organized in a solid pattern with oval to spindle nuclei and stippled chromatin. Although these features are not characteristic of PTC, the solid and spindle areas were misdiagnosed as poorly differentiated areas of PTC (Figure 2). Thus, this draws the conclusion that the presence of such a pattern should instigate the differential diagnosis of SETTLE and SS.

Because there were no obvious nuclear features of PTC, performing IHC would is advisable to confirm the diagnosis, which was performed in our case. On IHC, the tumor was found to be negative for thyroglobulin, which excludes the diagnosis of PTC (Figure 3a). In addition, the tumor was positive for HMWCK (34BetaE12) in both spindle cells and glandular cells (Figures 3b, 3c). Moreover, the tumor cells were found to be focally positive for CD99 (Figure 3d) but negative for chromogranin and BCL-2 (Figures 3e, 3f). The presence of such biphasic morphology with negative thyroglobulin staining and positive staining of AE1/AE3, HMWCK (34BetaE12), and CD99 (focal) confirms the diagnosis of SETTLE in our case.

Although there are several differential diagnoses for SETTLE, the literature substantiates that the biphasic SS and ectopic cervical thymoma majorly mimics SETTLE upon IHC [7,11]. The striking difference between SS and SETTLE is observed in the staining of cytokeratins and EMA positivity. Cytokeratin staining in SETTLE is strong and diffuse, whereas it is patchy in case of SS. Another differentiating factor is the indolent course of SETTLE. Similarly, EMA positivity is a reliable marker for SS as it is negative in SETTLE. Ectopic cervical thymoma is differentiated by the presence of lymphocytes mingled with the tumor cells [3].

Thus, the striking feature of SETTLE includes a predominantly cellular biphasic pattern characterized by a mixture of spindle and epithelial cells arranged in a glandular pattern.

## Conclusions

The rarity of this tumor and the reduced awareness regarding this tumor make it a diagnostic challenge. From our case report, it is evident that in case of any biphasic tumor (papillary and glandular component) of thyroid origin, which is often diagnosed as papillary carcinoma, a differential diagnosis of SETTLE should be considered. Thus, pathologists are encouraged to use IHC judiciously to rule out other mimics and establish an accurate diagnosis to help further treatment.
